# Cognitive Level Enhancement through Vision Exams and Refraction (CLEVER): study protocol for a randomised controlled trial

**DOI:** 10.1186/s13063-025-08813-x

**Published:** 2025-03-28

**Authors:** Srinivas Marmamula, Suvarna Alladi, Keerthana Umapathy, Ving Fai Chan, Graeme MacKenzie, Lynne Lohfeld, Asha Latha Mettla, Sridevi Rayasam, Vijaya K. Gothwal, Raja Narayanan, Giridhar Pyda, Harithaa P. Chadalavada, Priya Thomas, Lovemore Nyasha Sigwadhi, Augusto Azuara-Blanco, Cliona McDowell, Susan McMullan, Lynn Murphy, Mike Clarke, Joshua R. Ehrlich, Bonnielin Sweenor, Ciaran O’Neill, Shashidhar Komaravolu, Pallab K. Maulik, G. V. S. Murthy, Keshav Kumar, Anant Nyshadham, Achyuta Adhvaryu, Christopher McCabe, David E. Bloom, Jinkook Lee, Frank Lin, Seán Coghlan, Rohit C. Khanna, Nathan Congdon

**Affiliations:** 1 Allen Foster Community Eye Health Research Centre, Gullapalli Pratibha Rao International Centre for Advancement of Rural Eye Care, L V Prasad Eye Institute, Hyderabad, India; 2 Brien Holden Institute of Optometry and Vision Science, L V Prasad Eye Institute, Hyderabad, India; 3 Wellcome Trust/Department of Biotechnology India Alliance, L V Prasad Eye Institute, Hyderabad, India; 4 School of Optometry and Vision Science, University of New South Wales, Sydney, Australia; 5 National Institute of Mental Health and Neurosciences (NIMHANS), Bengaluru, India; 6 Present Address: Centre for Public Health, Queen’s University Belfast (QUB), Belfast, UK; 7 Riemann Limited, London, UK; 8 Clinical Trials Unit, L V Prasad Eye Institute, Hyderabad, India; 9 Patient-Reported Outcomes Unit - Brien Holden Eye Research Centre, L V Prasad Eye Institute, Hyderabad, India; 10 Northern Ireland Clinical Trials Unit (NICTU), Queens University Belfast (QUB), Belfast, UK; 11 Institute for Social Research, University of Michigan, Ann Arbor, MI USA; 12 Johns Hopkins University, Baltimore, MD USA; 13 Alzheimers and Related Disorders Society of India (ARDSI), Hyderabad, India; 14 George Institute for Global Health, New Delhi, India; 15 PRASHO Foundation, Hyderabad, India; 16 ICEH, London School of Hygiene and Tropical Medicine, London, UK; 17 University of Michigan, Ann Arbor, MI USA; 18 Department of Global Health and Population, Harvard T.H. Chan School of Public Health, Boston, MA USA; 19 University of Southern California, Santa Monica, CA USA; 20 Cochlear Center for Hearing and Public Health, Johns Hopkins Bloomberg School of Public Health, Baltimore, MA USA; 21 Brien Holden Eye Research Centre, L V Prasad Eye Institute, Banjara Hills, Hyderabad, India; 22 School of Medicine and Dentistry, University of Rochester, Rochester, NY USA; 23 Zhongshan Ophthalmic Centre, Sun Yat-Sen University, Guangzhou, China; 24 Orbis International, New York, NY USA; 25 Division of Epidemiology and Biostatistics, Department of Global Health, Faculty of Medicine and Health Sciences, Stellenbosch University, Cape Town, South Africa; 26 University of New South Wales, Sydney, Australia; 27 Department of Ophthalmology and Visual Sciences, University of Michigan, Ann Arbor, USA; 28 School of Medicine, Queen’s University Belfast (QUB), Belfast, UK; 29 School of Medicine, Dentistry and Biomedical Sciences, Queen’s University Belfast (QUB), Belfast, UK

**Keywords:** Cognitive decline, Correctable vision loss, Dementia, Elderly, LASI-DAD, Randomised controlled trial, India

## Abstract

**Background:**

Longitudinal observational studies have found an association between vision impairment and accelerated decline in cognition. However, no randomised trials have assessed the possible benefit of vision correction on cognitive change. We present the protocol for a three-year randomised controlled trial designed to assess the impact of spectacles for distance and/or near vision correction on cognitive change among community-dwelling elderly participants in India.

**Methods:**

Cognitive Level Enhancement through Vision Exams and Refraction (CLEVER) is a single-centre, open-label, parallel-group, individually-randomised trial. Participants (760 total, 380 in each arm) aged ≥ 60 years with correctable vision impairment at distance and/or near (presenting visual acuity < 6/18 in the better-seeing eye and improving to > = 6/18 with spectacles and/or presenting near vision worse than N6 at 40 cm and improving to N6 with spectacles), normal hearing (able to repeat at least three out of six words whispered from a 50 cm distance in the better ear) and normal cognition (Hindi Mini-mental Status Examination score > 18/31) will be enrolled. After a comprehensive eye examination, intervention group participants will receive distance, near, or bifocal spectacles, while controls will receive a prescription and spectacles at the end of the trial. The primary outcome will be the three-year change in Longitudinal Aging Study in India–Diagnostic Assessment of Dementia (LASI DAD) global cognitive factor score, with and without adjustment for baseline score, age, gender, education and other potential confounders.

**Conclusion:**

CLEVER is designed to assess the effectiveness of spectacles as a low-cost intervention to prevent or delay cognitive decline.

**Trial registration:**

This trial is registered with ClinicalTrials.gov, number NCT05458323, February 15, 2023.

**Supplementary Information:**

The online version contains supplementary material available at 10.1186/s13063-025-08813-x.

## Background

Dementia is a major health problem, affecting more than 57.4 million people globally [1]. By 2050, dementia or cognitive impairment will affect an estimated 152 million persons worldwide [1]. The increase in prevalence is greater in low- and middle-income countries (LMICs) such as India, because of relatively faster ageing and increases in life expectancy [2, 3]. Dementia increases the risk of mortality and adversely affects individuals and families [4, 5]. The global cost of dementia care is predicted to exceed US$2 trillion by 2030 [6]. The annual per capita cost for dementia care in LMICs ranges from US$590 for mild cases to US$25,500 for severe ones [7]. A substantial economic burden for dementia care is also reported in India [8]. The Alzheimer’s and Related Disorders Society of India estimated the total societal cost of dementia to be US$3.4 billion in 2010, rising to 0.5% of India’s gross domestic product by 2050 [9].

Vision impairment (VI) is also an important public health issue, affecting a billion people worldwide [10]. It is more prevalent in individuals aged ≥ 60 years, affecting one out of every three older adults in India [11]. Vision impairment adversely affects quality of life and visual functioning [12]. Moreover, it results in substantial economic productivity loss [13]. Approximately 80% of VI can be addressed with low-cost interventions such as spectacles and/or cataract surgery [10]. A recent RCT among Indian tea pickers reported a 22% increase in productivity when spectacles were provided [14].

Both dementia and VI are more common in elderly persons [15]. In India, the population aged > = 60 years is estimated to rise from 8% in 2015 to 19.1% in 2050 [16, 17]. Significant increase in the prevalence of both dementia and VI are expected to accompany this demographic change [15]. Therefore, research to identify modifiable risk factors and low-cost interventions to slow cognitive decline and prevent mild cognitive impairment, a potential precursor to dementia, is particularly important for India.

Observational studies have consistently found a positive association between vision impairment and cognitive decline [18]. The hypothesized pathways through which VI may influence cognitive health include increases in social isolation, physical inactivity, depression and changes in brain structure and increased cognitive load [5]. However, only experimental interventional study designs, such as randomised controlled trials (RCTs), can establish a cause-and-effect relationship between vision care and cognitive change. No such trials have yet been done.

The Cognitive Level Enhancement through Vision Exams and Refraction (CLEVER) trial aims to assess the impact of spectacles for distance and/or near vision correction on cognitive change among community-dwelling elderly persons in Telangana, India, over three years. This trial also seeks to understand the cost-effectiveness and impact of near and distance spectacles on quality of life, falls, depression, social interaction, and physical activity among study participants. The current report describes the CLEVER trial protocol.

## Methods

### Ethics approval and governance

The Institutional Review Boards (IRBs) of the Hyderabad Eye Research Foundation, LV Prasad Eye Institute (LVPEI [LEC-BHR-P-09–746]), the National Institute of Mental Health and Neurosciences (NIMHANS) [NIMHANS/ 36th 1EC (BS & NS DIV.Y)/2022], and Queen’s University Belfast (QUB) [MHLS22-13] have approved the trial protocol. Written informed consent will be obtained from all participants before enrolment into the trial. CLEVER will be conducted following the tenets of the Declaration of Helsinki and regulations set forth by governing bodies including the Indian Council of Medical Research (ICMR). The Health Ministry’s Screening Committee clearance for projects involving international collaborations has also been obtained as mandated by the ICMR.

The following oversight committees are in place to ensure rigorous monitoring of trial progress, including safety, efficacy, protocol adherence, and compliance with ethical, regulatory, and sponsor requirements:

### Trial Management Group (TMG)

The TMG oversees day-to-day trial management. It meets fortnightly, and includes the Chief Investigator (CI), local lead, collaborators, representatives from the Northern Ireland Clinical Trials Unit (NICTU), LVPEI Clinical Trials Unit (LVPEI CTU), project manager, qualitative lead, and research students.

### Trial Steering Committee (TSC)

An independent TSC acts on behalf of the sponsor and meets at least annually to oversee conduct of the trial. The CLEVER TSC comprises six independent members with expertise in public health, psychiatry, geriatrics, and study designs, alongside a Patient and Public Engagement (PPI) representative to ensure participant-centred perspectives.

### Data Monitoring and Ethics Committee (DMEC)

This multidisciplinary committee includes trialists, statisticians, public health specialists, ophthalmologists, and one cognitive neurologist. The DMEC meets at least annually, with meeting frequency adjusted as necessary. It evaluates participant safety, intervention efficacy, and trial conduct, providing recommendations to the TSC, sponsor, funder, and CI regarding trial continuation or modification.

Additionally, the LVPEI CTU, in coordination with the NICTU, conducts regular trial monitoring and audits as delegated by the sponsor, Queen’s University Belfast. Onsite visits are conducted at least quarterly, with additional visits as required to ensure compliance with the approved protocol, Good Clinical Practices (GCP), and data accuracy.

Management of protocol amendments will comply with the requirements of the sponsor (Queen’s University Belfast and their Research Ethics Committee [REC]), local IRBs (L V Prasad Eye Institute and NIMHANS), and the Indian Council of Medical Research (ICMR). Substantial amendments will require prior approval from QUB REC and LVPEI IRB, except in cases of urgent safety measures. Amendments will also be reported to ICMR in an annual progress report. Approved protocol amendments will be distributed by the LVPEI CTU to the research team for inclusion in the Investigator Site File. The team will be trained on these amendments, with documentation completed before implementation.

CLEVER is registered with ClinicalTrials.gov and the Clinical Trials Registry-India, and both registries will be updated with approved amendments. Any protocol deviations will be documented using a deviation/breach form, including an explanation and corrective/preventive actions. Deviations will be reported to QUB REC, local IRBs, and ICMR as per their standard procedures and in the annual progress report.

### Trial design

CLEVER is a single-centre, open-label, parallel-group, individually-randomised controlled trial, designed to test the impact of immediate versus delayed provision of free spectacles on rates of cognitive change among community-dwelling elderly persons.

### Participants

**Fig. 1:**
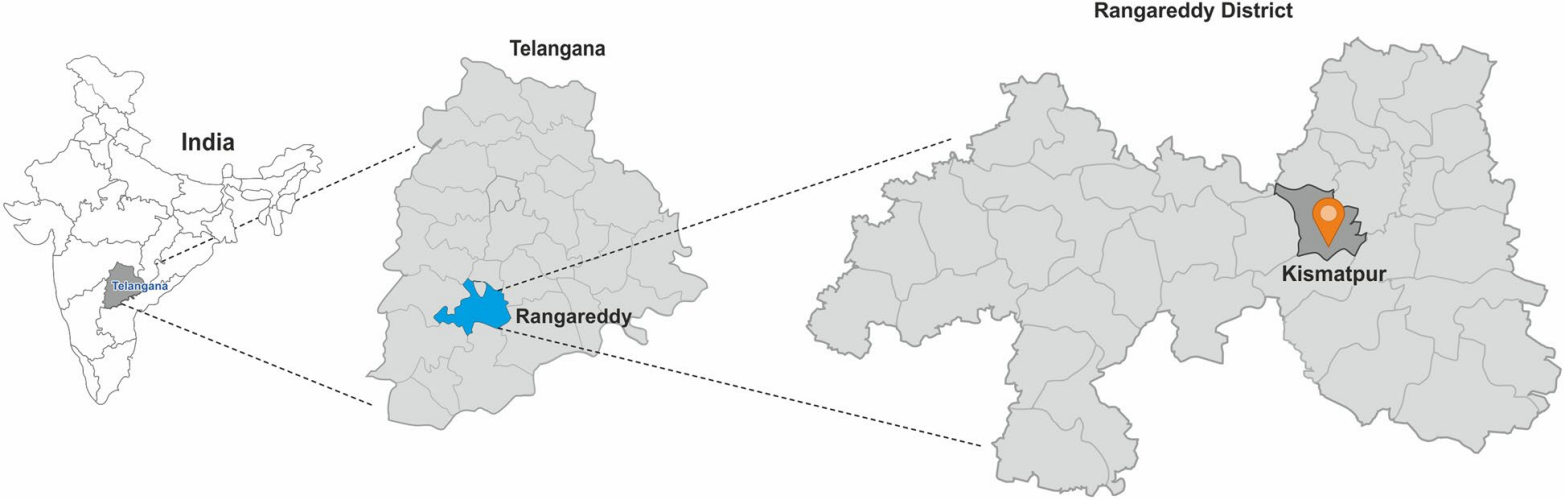
Map showing the study location (per-urban and rural location in Rangareddy district in Telangana state in India)

Community-dwelling elderly persons aged ≥ 60 years at the time of enumeration will be recruited from subdistricts in Ranga Reddy district in the South Indian state of Telangana (Fig. 1), according to the following criteria:

### Inclusion

1. Age ≥ 60 years at the time of enumeration.
2. Resident in the household for > = 3 months and planning to reside in the local area for the trial duration.
3. Distance vision impairment (VI, presenting visual acuity [VA] < 6/18 in the better-seeing eye and improving to > = 6/18 with spectacles) and/or near VI (presenting near vision < N6 at 40 cm and improving to > = N6 with spectacles).
4. Independent mobility with or without the support of a walking stick.
5. Hindi Mini-mental Status Examination (HMSE) questionnaire score > 18 (out of 31) [19].
6. Willingness to participate, to be randomised to either study group, and to adhere to the protocol.

### Exclusion criteria

1. Serious medical illness likely to result in loss to follow-up. Those less severely affected by conditions such as hypertension and/or diabetes will be eligible [20].
2. Failure on the whispered voice hearing screening test in the better ear (unable to repeat > = 3 out of 6 words whispered from behind the participant at a distance of 50 cm) [21, 22].

### Sample size

Based on the three-year, un-intervened decline in LASI-DAD (Longitudinal Aging Study in India–Diagnostic Assessment of Dementia) global cognitive score from a cross-sectional study of similarly aged persons in India [23, 24], with 90% power at *p* = 0.05 (two-tailed) using a two-sample t-test and estimated annual follow-up loss of 13% based on our other studies in this demographic group in the region, 760 participants across the two study groups are sufficient to detect a 29% effect size. Using a conservative estimate that 10% of screened persons will be eligible, an estimated 7600 will need to be screened to recruit 760 participants.

### Recruitment

**Fig. 2:**
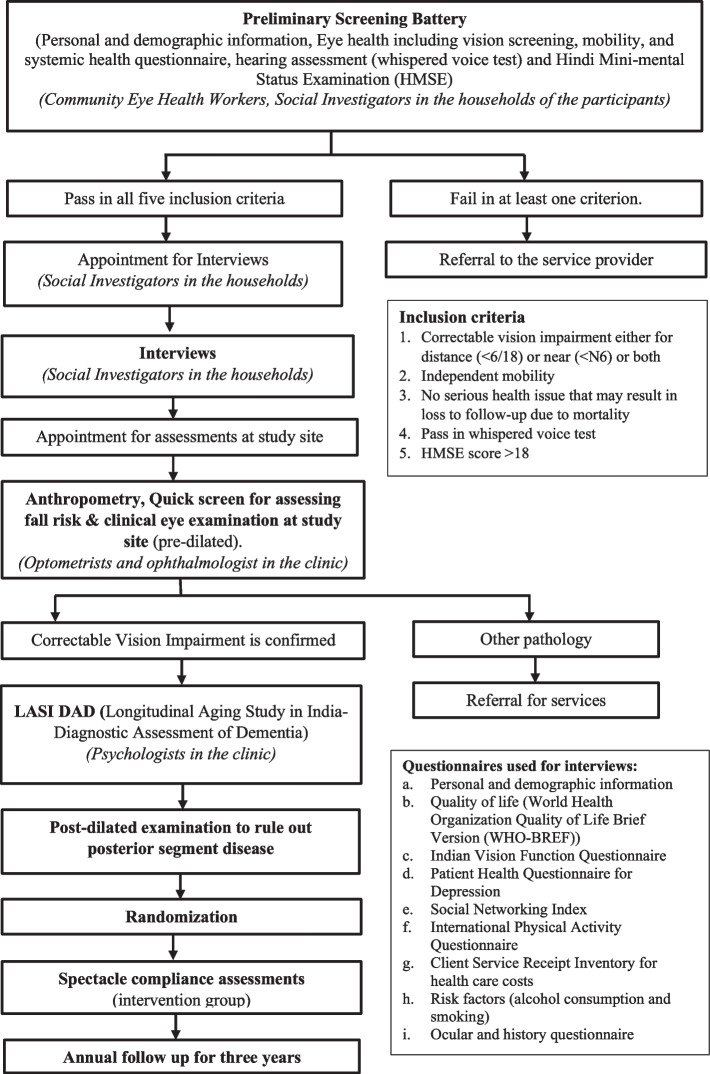
Study flowchart showing various stages of non-clinical and clinical assessment

Participants will be recruited through door-to-door household visits. Trained community eye health workers and social investigators will obtain written informed consent for preliminary screening after explaining study procedures. Each consenting participant will be assigned a unique identification number. The preliminary screening battery will include the following five components (Fig. 2):

1) vision screening to identify correctable VI at distance and/or near. Improvement in distance vision with pinhole is considered correctable VI (which will be confirmed with refraction in the clinical exam); 2) Ascertainment of independent mobility status, 3) Systemic medical history; [20] 4) hearing screening [21, 22]; and 5) screening for cognitive impairment using HMSE [19].

Social investigators will obtain written informed consent from all those eligible on the preliminary screening battery. Participants will be interviewed in their households and given appointments to visit the study clinic at the LVPEI centre (Shirin Etian & Tara Brown Eye Centre) in Kismatpur village, Ranga Reddy District, Telangana, for baseline clinical examination and cognitive testing [25, 26]. Those unable to visit the study site will be examined in temporary clinics set up in their vicinity.

### Primary outcomes

Global cognitive factor scores, the primary outcome measure, will be calculated using cognitive test data from the LASI-DAD battery, administered at baseline, and 12, 24, and 36 months later. The LASI-DAD battery of cognitive and neuropsychological tests assesses several domains of cognitive function and has been validated for use in India, including in Telangana [25, 26].

### Secondary outcomes

These include 36-month change in quality of life using the World Health Organization Quality of Life Brief Version (WHOQOL-BREF) [27], visual functioning (21-item Rasch version of the Indian Vision Function Questionnaire) [28], depression (Patient Health Questionnaire-9) [29], physical activity (International Physical Activity Questionnaire) [30], social interaction and networking (Social Networking Index questionnaire) [24], and falls risk (Quick Screen falls assessment) [31]. The trial will also assess the cost-effectiveness of the intervention as total intervention cost per quality-adjusted life years gained in the intervention group. The Client Service Receipt Inventory will be used to assess the resource use for medical care by the study participants [32] (Table 1).

**Table 1:** Schedule of assessments with details on location, personnel, and frequency

	Instrument name	Details	Baseline	Year 1	Year 2	Year 3
1	Personal and demographic information questionnaire	It includes age/gender, level of education, marital status, and income	✓			
2	World Health Organization Quality of Life Brief Version (WHOQOL-BREF) [27]	It has 26 items and covers four domains of quality of life: a) physical health, b) psychological well-being, c) social relationships, and d) environment	✓	✓	✓	✓
3	Indian Vision Function Questionnaire [28]	It has 20 questions covering visual functioning and mobility domains	✓	✓	✓	✓
4	Patient Health Questionnaire (PHQ9) for depression [29]	It has nine questions with scores of 0–3 points per question, for a total score of 0–27 points	✓	✓	✓	✓
5	Social Networking Index questionnaire [24]	It has 12 questions that assess the number of high-contact roles (network diversity), number of people in social networks, and embedded networks	✓	✓	✓	✓
6	International Physical Activity Questionnaire (IPAQ) Short form [30]	It has 7 questions and measures a 7-day recall of physical activities	✓	✓	✓	✓
7	Risk factors (alcohol consumption and smoking)	Includes questions on present and past smoking and alcohol consumption	✓	✓	✓	✓
8	Hindi Mini mental State Examination (HMSE) [19]	It is a 22 questions-based scale with a maximum score of 31	✓			
9	Ocular history and systemic health questionnaire	It includes questions on previous surgeries, use of spectacles, and duration of use. Systemic health questionnaire includes information on hypertension, diabetes and other non-communicable diseases	✓	✓	✓	✓
10	Longitudinal Aging Study in India–Diagnostic Assessment of Dementia; LASI-DAD cognitive test battery [25, 26]	It covers the key cognitive domains: memory, attention/speed, orientation, language function, visuospatial skills, executive function, numerical ability, and retrieval fluency	✓	✓	✓	✓
11	Client Service Receipt Inventory for healthcare costs [32]	This collects information on the whole range of services, including estimating the costs of service receipt	✓	✓	✓	✓
12	Clinical Falls Risk Assessment: Quick screen questionnaire [31]	It assesses five factors related to falls: history of falls in the last 12 months, regular use of four or more medications, vision, peripheral sensation, balance, time reaction, and lower limb strength	✓	✓	✓	✓

### Study team

A multidisciplinary team, including vision care professionals (ophthalmologist, optometrists and vision technicians), neuropsychologists and psychologists, social investigators, and community eye health workers will conduct this trial. The logistics of the trial, including appointments, management of the trial site, treatment allocation based on clinical trial unit (CTU) recommendation, and tracking of spectacles compliance, will be organized by a trial coordinator. The principal investigators (NC, RCK) are responsible for the scientific and administrative aspects of the trial, with the support of the TMG.

### Interviews

Trained investigators will conduct personal interviews in the participant’s native language (Telugu or Hindi). Adequate time will be allotted for participants to rest between assessments. The same investigator will conduct the entire interview for each participant.

### Clinical examination

This includes the following components, 1) external examination (including ocular motility and alternate cover test); 2) assessment of monocular near (using a logarithm of minimum angle of resolution [logMAR] chart at 40 cm) and distance visual acuity (VA, unaided, best-corrected, pinhole, and with existing spectacles if available, with a logMAR chart at 3 m using tumbling E optotypes, at an illumination of ≥ 180 lx measured with a light meter); 3) objective and subjective refraction, for participants with presenting distance VA worse than 6/9 or near VA worse than N6 in either eye; 4) slit-lamp biomicroscopy and assessment of the lens status after pupil dilation (SL 120 Carl Zeiss Meditec, Inc., Dublin, CA); 5) applanation tonometry (Carl Zeiss Meditec, Inc., Dublin, CA); 6) fundus examination and imaging with pupil dilation. In addition, seven field fundus photographs (Centervue, Italy) and lens photography with optical section and retro illumination view on a slit lamp camera (Haag-Streit Imaging module 910, Haag-Streit, Switzerland) will be captured. Retinal Optical Coherence Tomography (Cirrus Optical Coherence Tomographer, Carl Zeiss Meditec, Jena, Germany), with 5-line raster and macular cube will be performed on all participants.

Serum haemoglobin A1C (HbA1C) levels will be assessed on all participants (NycoCard reader, Abbott Diagnostics Technologies AS, Oslo, Norway) to assess blood glucose control over recent months. Inter-examiner reliability will be assessed for measurement of VA and refraction. Pilot testing of all examination procedures will be carried out with 20 eligible participants before beginning the main trial.

### Cognitive examination

The complete LASI-DAD neuropsychological test battery will be performed in the Telugu or Hindi languages. It assesses the following domains, 1) Orientation (Community Screening Instrument for Dementia); 2) visuospatial (constructional praxis); 3) language fluency (retrieval fluency); 4) immediate and delayed memory (word recall); 5) executive functions (Go-No-Go task); and 6) decision-making and judgment (judgement and problem-solving) [25, 26]. Psychologists will be trained to administer the cognitive test battery on patients and healthy older adults at the National Institute of Mental Health & Neuro Sciences (NIMHANS) under the supervision of the Clinical Psychologist/Neuropsychologist before performing the assessments on participants in the pilot and main trial.

### Intervention

All participants in the intervention group will receive free near and/or distance spectacles plus instructions on their use within two weeks of the examination, with annual replacement in case of loss, breakage or change in power such that VA falls below the enrolment criteria. Adherence with use of spectacles will be assessed in both study groups through phone calls made every three months and half yearly home visits. Intervention group participants adherent with spectacle wear will be rewarded through nonfinancial incentives such as vouchers for diagnostic tests and other utility items at regular intervals. There will be no special criteria for discontinuing or modifying allocated interventions. No attempts will be made to prevent control participants from obtaining spectacles outside the study based on the prescriptions provided.

### Controls

All participants randomised to the control group will receive a prescription for spectacles at baseline, as is the standard of care in the area, and will receive near and/or distance spectacles at no cost at study closeout.

### Randomisation, allocation and masking

The randomisation sequence will be generated by the trial statistician in the LVPEI CTU using an online randomisation tool (https://www.sealedenvelope.com). Stratified randomisation will be done based on gender (male/female), age (below and above median), education level (below and above median), and baseline HMSE (below and above median). Group allocation will be concealed from the trial coordinator and field team until a potential participant is determined eligible. Trial personnel will not be masked to participants’ treatment assignment due to practical difficulties in assessing the main trial outcome without study spectacles being worn. Masking of participants with use of zero-power spectacles among controls is not considered ethical in this setting.

### Monitoring and quality control

Adherence to study protocols will be monitored by random visits to the clinic sites and the communities where preliminary screening takes place. The investigators will review all case report forms before data entry to ensure completeness. Protocol deviations, defined as variations from the IRB-approved protocol, will be fully documented, including corrective measures taken, and will be reported to the DMEC and the IRBs approving the study protocol.

All team members are trained thoroughly on study procedures, as documented by the training log. Investigators visit the field biweekly to review the performance of staff. Retraining and orientation are provided every six months.

## Data management and analysis plan

Data will be collected on paper forms initially and then entered into an electronic database developed in Microsoft Access (Microsoft Corporation, Redmond, Washington, USA) at the data centre at Gullapalli Pratibha Rao International Centre for Advancement of Rural Eye care in Hyderabad, Telangana, India. Data will be securely stored in encrypted cloud-based institute servers. Personal data will be stored in a different database with access restricted to primary investigators. Data will be analysed at the end of the trial when all follow-up assessments are completed. All randomised participants will be assessed based on the intention-to-treat principle in the main analysis. Per-protocol analysis will also be conducted as secondary analysis, analysing participants according to treatment they actually received. No formal interim analysis will be done for the primary outcome measure.

Baseline characteristics and follow-up measurements will be summarised as mean and standard deviation, median and inter-quartile range, or numbers and frequencies (%) as appropriate, depending on the scale and distribution of measurements. Histograms or Q-Q plots will be used to assess the normality of all continuous variables.

Global cognitive factor scores for our study population will be calculated using the cognitive test data from the LASI-DAD battery. This calculation is similar to that performed in the ACHIEVE trial, which investigated the impact of hearing aids on cognitive decline [33]. In ACHIEVE, the global cognitive factor score was the primary outcome measure calculated using the ARIC (Atherosclerosis Risk in Communities) cognitive battery [33]. The between-group difference in three-year change in LASI-DAD global cognitive score (primary outcome) will be calculated. A linear mixed-model regression analysis with restricted maximum likelihood estimation will be used to assess between-group differences in change in LASI-DAD global cognitive score over time after adjusting for potential confounders (baseline cognitive score, age, sex, education, visual function, quality of life, depression, falls, social interaction, and physical activity).

Three-year change from baseline in quality of life, visual functioning, depression, falls, social interaction and networking, and physical activity will be the secondary outcomes. Rasch analysis will be conducted to derive interval scores from quality of life and vision-related quality-of-life instruments. A mixed-effects binomial regression with a log link will be used to estimate the relative risk, and a binomial model with an identity link for estimating the risk difference will be used to assess between-group differences in change in depression. A mixed-effects linear regression with an identity link will be used to estimate the between-group mean difference in change in falls. Participants will be placed into three categories of physical activity and social interaction for these analyses as previously described [30, 34].

The cost-effectiveness analysis will estimate the expected incremental cost per unit difference in the cognitive factor scores between the intervention and the control groups. A health and social care perspective will be used for the analysis. A Markov model will be developed, allowing the comparison of the CLEVER intervention with alternative interventions for preventing cognitive decline. An “in-trial” analysis that compares the costs and outcomes of the two study groups for the time horizon of the trial will be conducted. Multinomial logistic regression will be used to adjust for potential confounders. Odds ratios and their 95% confidence intervals will be reported.

Statistical significance of all results will be assessed at the conventional level of *p* < 0.05 (two-tailed). Stata (V.16, Stata Corp, College Station, Texas, USA) will be used for all statistical analyses.

## Discussion

CLEVER is the first trial anywhere to investigate the potential cause-and-effect relationship between provision of spectacles and the rate of cognitive decline in an elderly population. Spectacles are one of the most cost-effective interventions in all of health care, and uncorrected near and distance refractive error are the most common cause of VI world-wide. If spectacles are proven to slow cognitive decline, then service delivery programs can be developed to address the twin public health problems of VI and cognitive impairment. The impact on the health of an aging global population will be substantial, especially for the 70% living in LMICs, who currently lack proven, low-cost treatments to delay or prevent dementia.

The multidisciplinary team of specialists, including ophthalmologists, optometrists, cognitive neurologists, social investigators, and neuropsychologists is an added strength of the trial.

## Conclusions

The randomised, controlled design of CLEVER will provide robust evidence for the impact of low-cost vision care on cognitive health. The information gained from a positive trial result would contribute significantly to the goal of healthy aging in India and other LMICs.

## Supplementary Information

Supplementary Material 1

## Data Availability

Not applicable.

## Abbreviations

CLEVER: Cognitive Level Enhancement through Vision Exams and Refraction
CSRI: Client Service Receipt Inventory
CTU: Clinical trial unit
DMEC: Data Monitoring and Ethics Committee
logMAR: Logarithm of minimum angle of resolution
LASI-DAD: Longitudinal Aging Study in India-Diagnostic Assessment of Dementia
LVPEI: L V Prasad Eye Institute
HbA1C: Haemoglobin A1C
HMSE: Hindi Mini-mental Status Examination
IPAQ: International Physical Activity Questionnaire
LMICs: Low- and middle-income countries
NIMHANS: National Institute of Mental Health and Neurosciences
OCT: Optical Coherence Tomography
PHQ9: Patient Health Questionnaire-9
QUB: Queen’s University Belfast
SNI: Social Networking Index
VA: Visual acuity
VI: Vision impairment
WHOQOL-BREF: World Health Organization Quality of Life Brief Version
